# Rhizospheric *Bacillus*-Facilitated Effects on the Growth and Competitive Ability of the Invasive Plant *Ageratina adenophora*

**DOI:** 10.3389/fpls.2022.882255

**Published:** 2022-06-14

**Authors:** Ewei Du, Yaping Chen, Yahong Li, Zhongxiang Sun, Furong Gui

**Affiliations:** ^1^State Key Laboratory for Conservation and Utilization of Bioresources in Yunnan, College of Plant Protection, Yunnan Agricultural University, Kunming, China; ^2^Yunnan Plant Protection and Quarantine Station, Kunming, China

**Keywords:** *Ageratina adenophora*, invasive plant, rhizosphere, *Bacillus*, competitive advantage, plant-soil feedback

## Abstract

The rhizospheric microbial community affects the population establishment of invasive plants in introduced areas, among which *Bacillus* has numerous functions in promoting plant growth. This study isolated and enriched the *Bacillus* community in the rhizospheric soil of the invasive plant *Ageratina adenophora* and the native accompanying plant *Rabdosia amethystoides*. The effects of these rhizospheric *Bacillus* communities on the growth and competition of *A. adenophora* and *R. amethystoides* were evaluated in pot experiments. The results showed that the number and diversity of *Bacillus* in the rhizospheric soil of *A. adenophora* were higher than those of *R. amethystoides* (*A. adenophora*: 122 strains in soil, 16 *Bacillus* taxa; *R. amethystoides*: 88 strains in soil, 9 *Bacillus* taxa). After *Bacillus* inoculation of *A. adenophora* in a pot experiment, *Bacillus idriensis*, *Bacillus toyonensis* and *Bacillus cereus* were accumulated in the rhizospheric of *A. adenophora*, which significantly increased the nitrate nitrogen (NO_3_^–^-N) content in the soil and the total carbon and nitrogen concentrations in *A. adenophora* in the mixed treatment. The selective accumulation of *Bacillus* enhanced the competitive advantage of *A. adenophora* over the native accompanying plant; the corrected index of relative competition intensity of *A*. *adenophora*-inoculated *Bacillus* reached double that of the uninoculated treatment, and the growth of native plants was greatly suppressed under mixed planting. Our study confirmed that invasion of *A. adenophora* can lead to the accumulation of specific *Bacillus* taxa in the rhizospheric soil, which in turn can increase the competitive advantage of *A. adenophora*.

## Introduction

Invasive weeds are a major threat to the structure and function of global ecosystems (van der Putten et al., 2007; D’Antonio et al., 2017) and cause huge economic losses (Duncan et al., 2004). Understanding the invasion mechanism is crucially important to effectively control and develop management strategies for invasive weeds. Modifications in the microbial community induced by invasive plants have the potential to initiate a self-promoting mechanism that facilitates the invasion process (Callaway and Ridenour, 2004; Massenssini et al., 2014; Fang et al., 2019). For instance, such changes in the microbiota associated with nutrient cycling induced by the exotic plant *Conyza canadensis* may have a beneficial effect that promotes its establishment and spread (Zhang et al., 2020). Beneficial microorganisms, such as arbuscular mycorrhizal (AM) fungi, plant growth-promoting rhizobacteria (PGPR), and rhizobia, were accumulated in the rhizosphere of invasive plants during their growth process, promoting their growth and competitiveness (Rodríguez-Echeverría, 2010; Philippot et al., 2013; Zhang et al., 2017). In the rhizospheric soil of the invasive plant *Flaveria bidentis*, the levels of diazotrophs, phosphorus-solubilizing bacteria, and silicate-solubilizing bacteria were much higher than those of native plants (Song et al., 2016, 2017). Compared with native plants, the invasive plants *Avena barbata* and *Bromus secalinus* increase the number of ammonia-oxidizing bacteria in soil, improve soil nitrogen cycling, and increase the soil nitrogen content, enhancing the growth and competitiveness of invasive plants (Hawkes et al., 2005). Consequently, invasive species can establish more positive feedback interactions with the soil microbiota and appear to have a greater dependence on these associations than noninvasive species (Massenssini et al., 2014; Trognitz et al., 2016).

The genus *Bacillus* has tremendous genetic and metabolic diversity, and they perform various ecological functions in the soil ecosystem, including nutrient cycling and endowing plants with stress tolerance (Choudhary and Johri, 2008; Dheeman et al., 2017). These bacteria can form endospores, allowing them to survive in hostile environments and to perform well under different environmental conditions (Gardner, 2004; Pesce et al., 2014). *Bacillus* strains can be used as plant growth promoters and biocontrol agents, promoting plant growth under various environmental conditions (Kumar et al., 2011; Saxena et al., 2020). The principal mechanism includes increasing the nutrient content and uptake by nitrogen fixation, phosphate, and potassium solubilization (Sharma et al., 2013; Ding et al., 2015; Verma et al., 2015), regulating plant hormone production (Alina et al., 2015; Asari et al., 2017), reducing abiotic and biotic stresses by biofilm formation, producing volatile organic compounds (VOCs), and inducing systemic resistance (Li et al., 2015; Burkhanova et al., 2017; Pandin et al., 2017). For example, *Bacillus pumilus* M8 significantly reduced the *in vitro* growth of *Botrytis cinerea* and *Fusarium solani* phytopathogens and induced systemic resistance of pepper to gray mold (Márquez et al., 2020). In both the presence and absence of salt stress, *Bacillus licheniformis* A2 increased peanut plant growth (Goswami et al., 2014). Several other *Bacillus* taxa such as *Bacillus megaterium*, *Bacillus cereus*, *Bacillus subtilis*, *B*. *licheniformis*, *Bacillus mycoides*, *Bacillus idriensis*, and *Bacillus vietnamensis* are known to promote plant growth by the main mechanisms described above (Goswami et al., 2016; Radhakrishnan and Lee, 2016; Yousuf et al., 2017; Fan et al., 2018). Therefore, studies on the relationship between *Bacillus spp.* and invasive plants are important to further elucidate the colonization and dominance of invasive plants in non-native habitats.

*Ageratina adenophora* (Sprengel, also known as *Eupatorium adenophorum* Sprengel) is a notorious weed that originated in Mexico and Costa Rica, invading more than 40 tropical and subtropical countries in Asia, Oceania, Africa, and Europe (Datta et al., 2019; Gu et al., 2021). It first invaded Yunnan Province, China, in the 1940s and is currently widely distributed in six provinces in Southwest China. It continues to spread to the east and north at a rate of approximately 20 km/year (Wang and Wang, 2006; Wan et al., 2010). It can reduce the diversity of native plant species, crop productivity, and forage production in pastures, resulting in severe economic losses for agricultural, forestry, and livestock industries (Gui et al., 2009; Kong et al., 2017; Poudel et al., 2019). An increasing number of evidence suggests that *A. adenophora* can alter the soil microbial community, promoting its growth and competitiveness while inhibiting neighboring native plants (Li et al., 2009; Xu et al., 2012; Zhao et al., 2019). For example, *A. adenophora* invasion leads to a decrease in actinomycetes but increases in aerobic, anaerobic bacteria, and nitrogen-fixing bacteria (Niu et al., 2007; Xu et al., 2012; Fang et al., 2019). The rhizospheric soil microbiomes of *A. adenophora* differed to varying degrees in the relative abundances of bacterial and fungal phyla and genera from those of two native plants, *Artemisia indica* and *Imperata cylindrica*, and were more metabolically active than both of these, as indicated by marked increases in the expression levels of genes associated with the cell wall, cell membrane, and envelope biogenesis, energy production and conversion, and the transport of carbohydrates, amino acids, coenzymes, nucleotides, and secondary metabolites (Xia et al., 2021). Previous studies have reported that *A. adenophora* can result in the accumulation of *Bacillus*, such as *B. megaterium* and *B. cereus*, which might significantly promote the growth of *A. adenophora* compared to native plants (Niu et al., 2007; Sun et al., 2021). Nonetheless, it remains unknown whether *A. adenophora* can selectively accumulate *Bacillus* from the local soil to promote its competitive growth. We hypothesized that the invasion of *A. adenophora* may involve the accumulation of *Bacillus* in its rhizosphere, increasing its competitiveness against native plants. To investigate this hypothesis, we analyzed and compared the composition and abundance of the culturable *Bacillus* community in the rhizospheric soil of the invasive plant *A. adenophora* and the native accompanying plant *Rabdosia amethystoides* and determined the effects of *Bacillus* on the growth of *A. adenophora* using a pot experiment.

## Materials and Methods

### Field Site and Sampling Approach

Soil sampling was conducted at two sites in the same habitat at Yunnan Agricultural University in Yunnan Province, China, in the summer of 2020 (Lat. 25°08′30′′N, Lon. 102°45′13′′E, with an elevation of 1,940 m). Site I was dominated by *A. adenophora*, whereas site II was dominated by *R. amethystoides*. The coverage of the two plants was higher than 80%, and the abiotic factors such as light and soil texture were basically the same. Each site had three plots with a distance of approximately 5 m between plots. A number of ten plants were randomly collected from plots of 5 m × 5 m. Soil samples were collected from each plot as follows: 1 cm of litter was removed from the soil surface, and underlying soil within a 30-cm radius of each plant was loosened with a shovel. Then, plants were dug out, lightly shaken, and the soil remaining attached to the root surface was carefully collected with a brush; approximately 150–200 g of soil was extracted from each plot. Because the plants grew in a relatively high density and thus might have a large impact on the surrounding soil, these soil samples were operationally defined as rhizospheric soils (Batten et al., 2006). The collected samples from the same plots of the same site were mixed to form two soil samples, representing the rhizospheric soil of *A. adenophora* and *R. amethystoides*. The soil was sieved through a 2-mm sieve, transferred to sterile plastic bags, cooled immediately, transported to the laboratory, and stored at 4°C. Each sample was assigned to two groups: one group was used for chemical analysis and the other for *Bacillus* community isolation and analysis.

### Isolation of *Bacillus*

*Bacillus* was explicitly enriched in the soil, and a combination of heat-shock and serial dilution was used to isolate *Bacillus*, as Kumar et al. (2012) described. Briefly, soil samples (10 g) from the rhizosphere of *A. adenophora* and *R. amethystoides* were heat-treated (80°C), transferred to 90 ml sterile distilled water, and mixed by shaking (180 rpm) the triangular flask for 12 h at 30°C. After serial dilution suspension to 10^–3^, one part was used to isolate and identify strains, and the other part was used to prepare inoculants for pot experiments. For the isolated strains, the aseptic dilution involved the collection of a 0.1 ml suspension using a micropipette that was streaked on nutrient agar plates in triplicate. The plates were incubated at 30°C for 48 h. The number of *Bacillus* was obtained by counting the colony-forming units (CFUs), and the data were expressed as CFU per gram dry weight of each soil sample. All isolates were collected and purified based on the quadrant method until a single colony was obtained. The single colony was selected by an aseptic toothpick and was separately incubated in a 1.5-ml centrifuge tube (1 ml nutrient liquid medium culture in a 1.5-ml centrifuge tube) that was shaken at 37°C (200 rpm) for 12 h. Some of the *Bacillus* in the centrifuge tube were dissolved in 50% glycerin (v:v = 1:1) and were stored at –20°C for short-term storage. With respect to the inoculants, a 4 ml suspension was placed in a triangular flask containing 100 ml nutrient liquid medium (1% peptone, 0.3% beef extract, 0.5% NaCl) that was shaken (180 rpm) for 24 h at 37°C. The optical density of the suspension was adjusted to approximately 1.0 (optical density at 600 nm) by diluting it with sterile distilled water. The population count of *Bacillus* was maintained at 10^8^ CFU/ml.

### Analysis of Culturable *Bacillus* Diversity

#### DNA Extraction

The isolated *Bacillus* culture suspension was centrifuged at 12,000 r/min for 10 min at 4°C. The suspension was agitated to homogeneity, and centrifugation was performed; the supernatant was removed, and 400 μl of 10 × TE and 25 μl of lysozyme (50 mg/L) were added to the tubes. The tubes were then shaken horizontally at 180 rpm for 12 h at 37°C. Then, 100 μl of 10% SDS was added, and the tubes were incubated in a 60°C water bath for 30 min and centrifuged as described above. Subsequently, the lysate was combined and mixed with an equal volume of phenol–chloroform–isoamyl alcohol (25:24:1). The supernatant was extracted with two times the volume of chloroform–isoamyl alcohol (24:1). The aqueous phase was precipitated with an equal volume of isopropanol at –20°C overnight. The aqueous phase was precipitated with an equal volume of isopropanol at –20°C overnight. The nucleic acid pellet was centrifuged at 12,000 *g* for 10 min at room temperature. The crude extracts were washed with 70% cold ethanol, resuspended in 50 μl of diethylpyrocarbonate-treated water, and stored at –20°C. The quality and quantity of DNA were detected by a NanoDrop 2000 ultramicro spectrophotometer (NanoDrop 2000, Thermo Scientific, United States).

### 16S rRNA Gene Amplification and Sequencing

Amplification of 16S rDNA by polymerase chain reaction (PCR) was carried out using the universal primers F27 (5′-AGAGTTTGATCCTGGCTCAG-3′) and R1492 (5′-GGTTACCTTGTTACGACTT-3′) (Weisburg et al., 1991). The polymerase chain reaction was performed in a 30-μl reaction volume containing approximately 20 ng of template DNA, 10 μm of each primer, 10 mM dNTPs, and 1.5 U Taq polymerase in 1 × PCR buffer. Reactions were cycled 30 times at 94°C for 1 min, 60°C for 45 s, and 72°C for 1 min, followed by a final extension at 72°C for 8 min. The amplified PCR product (1,500 bp) was separated by electrophoresis on 1% (w/v) agarose gels containing ethidium bromide and was visualized using a UV-transilluminator. Sequencing was conducted by Tsingke Biological Technology (Kunming, China). A BigDye Terminator kit (BDT v1.1) was used for the reaction; a 20 μl reaction volume contained 1 μl of DNA, 8 μl of BigDye, 1 μl of primer, and 10 μl of deionized water. Reactions were performed at 96°C for 1 min, (96°C for 10 s, 50°C for 5 s, and 60°C for 4 min) 25 times. Purification was performed by adding Magical Buffer and Ferrite Bead reagent followed by centrifugation. The DNA sequencing was performed using an automated DNA sequencer (ABI PRISM 3730XL Genetic Analyzer) (Applied Biosystems). Data were obtained from the sequencer and were analyzed by Sequencing Analysis 5.2 to form the final sequence.

### 16S rDNA Sequence Downstream Processing and Phylogenetic Analysis

The 16S rDNA sequence was assembled with DNAStar 6.0 and was added to the SeqMan program, where it was cut to eliminate 30 to 60-bp nucleotides at both ends of the sequence, and by clicking ‘‘Assemble,’’ homologous sequence linking was executed. Sequences were imported into the EZbiocloud software package^1^, and comparisons were carried out with BLAST homology compared with the known 16S rDNA sequence. 16S rDNA sequence similarity of ≥ 97% with a prototype strain sequence from the GenBank database was used for the identification of the species level. Multiple sequences and several near-margin sequences were aligned using the Clustal-W program in MEGA X software (version X, Mega Limited, Auckland, New Zealand). The phylogenetic tree was constructed using the neighbor-joining method (NJT), which was implemented in the MAGE X program (Kumar et al., 2018). The T92+G+1 (Tamura3-parameter and Gamma distributed) model was chosen as the best model for phylogenetic tree analysis and 1,000 bootstrap replications. One representative strain of each bacterium isolated and identified in this experiment was selected, and then, the nucleotide sequences of these strains were submitted to NCBI GenBank to obtain accession numbers (OM149778-OM149795). The relative abundance (RA) of *Bacillus* taxa in our total sample was calculated according to the following formula: RA = A/N × 100%. (Note: “A” represents the number of one *Bacillus* phylotype strains and “N” represents the total number of strains).

### Alpha Diversity of *Bacillus* Community

The Shannon (H’) index and Simpson’s diversity index (D) were calculated according to the formula as follows:


$$H^{\prime }=\sum_{i=1}^{s}P_{i}\,l\,n\,P_{i}$$

$$D=1-\sum {\left(N\,i/N\right)}^{2}$$

(“S” represents the total number of *Bacillus* phylotypes; P_i_ = N_i_ / N, “N_i_” represents the number of the *Bacillus* phylotype *i*, “N” represents the number of all *Bacillus* phylotypes).

### Soil *Bacillus* Number and Diversity After the Pot Experiment

The collected soil samples from the same inoculation and plant growth treatment were mixed to form six soil samples, representing Am, A+R, and Rm of two inoculation treatments. This was done for each soil sample using heat-shock and serial dilution of isolated *Bacillus*. Colony separation 10^–3^ of the supernatant was used to count the colony-forming units. All isolates were collected and purified. The identification of *Bacillus* strains, alpha diversity, and the relative abundance of *Bacillus* strains were carried out according to the above method.

### The Effect of *Bacillus* on the Competitive Growth of *A. adenophora*

A greenhouse experiment was carried out to test the effect of *Bacillus* from the rhizosphere of *A. adenophora* or *R. amethystoides* on the competitive growth of *A. adenophora* at Yunnan Agricultural University. On three successive days, soil without plant cover was autoclaved for 2 h at 121°C to remove soil microbes. The basic properties of the soil were as follows: pH (w/v water = 1:5) was 6.25, the organic matter content was 15.502 g/kg, total nitrogen was 0.899 g/kg, total phosphorus was 0.351 g/kg, total potassium was 40.03 g/kg, available nitrogen was 20.28 μg/g, available phosphorus was 5.089 μg/g, and available potassium was 32.32 μg/kg. The sterilized soil was used for three treatments: monoculture of *A. adenophora*, monoculture of *R. amethystoides*, and an equal mixture of *A. adenophora* and *R. amethystoides*. Each treatment was further divided into three levels: uninoculated treatment (C), inoculated with *Bacillus* from *A. adenophora* (AB), and inoculated with *Bacillus* from *R. amethystoides* (RB). Seeds of *A. adenophora* and *R. amethystoides* were obtained from Yunnan Agricultural University and were surface-sterilized in 1.5% sodium hypochlorite (NaClO), rinsed five times with sterile distilled water, submerged in 70% ethanol for 1 min, and then washed five times with sterile distilled water. A number of ten seeds of *A. adenophora* or *R. amethystoides* (or five seeds of the two plants in a mixture) were added to 1 kg of soil in the pots (20 cm × 13 cm × 14 cm for length × width × height) and were covered by approximately 1 cm of soil, and the bacterial suspension of *A. adenophora* or *R. amethystoides* was inoculated (10 mL 10^8^ CFU/ml). *Bacillus* suspension was added one time per month during the planting period for a total of four times. The plants germinated in approximately 5 days. Then, 10 days after germination, excess plants were removed, so that each pot contained only two of the same size plants (two monocultures, one from each of the mixed-cultures), with 10 replicates per treatment. Sterile water was applied every 2 days. The pots were placed on a shelf in the greenhouse for 4 months under a 14-h L:10-h D photoperiod at 28°C and were arranged in a completely randomized design.

### Measurements

#### Biomass and Corrected Index of the Relative Competition Intensity

All parameters were measured 4 months after sowing. Plants were dug out, and the soil attached to the root surface was collected. All plants were oven-dried at 80°C for 48 h before specific measurements were taken. Dry biomass was measured using the entire plant, including the above-ground biomass and root biomass. The corrected index of relative competition intensity (CRCI) was used to test the plant’s competitive ability. This index was calculated following the method of Oksanen et al. (2006).


CRCI=arcsin⁢[(X-Y)/max⁢(X,Y)],

where X is the average biomass without competition and Y is the average biomass of individual plants grown in competition. A CRCI value > 0 indicates that competition has a negative effect, and a CRCI value < 0 indicates that competition positively affects the target plant.

#### Total Nutrient Concentrations in the Plant

The concentration of C in the plants was measured by a CHN analyzer (LECO Corporation). The concentration of N was determined by the micro-Kjeldahl procedure (Nelson and Sommers, 1972). The concentrations of P and K were determined by inductively coupled plasma spectroscopy (Isaac and Johnson, 1983).

#### Soil Characteristics

After the plants were harvested, the characteristics of the potting soil were assessed. A Chem II flow-injection analyzer (QC8500S2, Lachat, United States) was used to quantify the contents of ammonium nitrogen (NH_4_^+^-N) and nitrate-nitrogen (NO_3_^–^-N) in KCl extracts (Li and Li, 2013). Available phosphorus (AP) was determined according to the methods described by Olsen (Olsen, 1954). The burnt-luminosity method was used to determine the content of available potassium (AK) (Lu, 2000).

#### Statistical Analysis

Before analyses, all data were tested for normality using the Shapiro–Wilk test. All data met the normality assumption. The variables in the treatments were expressed as the mean ± SE. One-way analysis of variance (ANOVA) was performed to determine the differences in biomass, nutrient concentrations of the plants (C, N, P, and K), and the available soil nutrient contents in different inoculated treatments and to determine the effect of *Bacillus* on the competitive potential (CRCI) of both species. Multiple comparisons between groups were performed using the least square differences (LSD). Student’s *t*-test was used to determine the effect of monoculture and the mixture on the plant growth parameters. Mean values and standard errors per treatment combination were presented (*n* = 5). All analyses were conducted using SPSS 19.0 (SPSS Inc., Chicago, IL, United States).

## Results

### *Bacillus* Community Structure in Different Treatments

#### *Bacillus* Community Structure in the Rhizospheric Soil of the Two Plants

**FIGURE 1:**
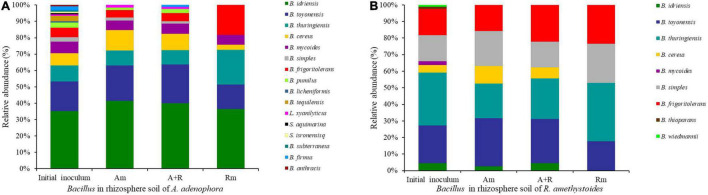
The relative abundance (RA) detected for each *Bacillus* phylotype in rhizospheric soil of *A. adenophora* **(A)** and **(B)** in different treatments. Am, *A. adenophora* monoculture; A+R, *A. adenophora* and *R. amethystoides* mixture; Rm, *R. amethystoides* monoculture.

A total of 122 sequences were obtained from *A. adenophora* rhizospheric soil. A number of twelve phylotypes belonged to the genus *Bacillus*, and four others belonged to the phylotypes of other genera in the Bacillaceae family (*Brevibacterium frigoritolerans*, *LysiniBacillus xylanilyticus*, *Sporosarcina aquimarina*, and *SoliBacillus isronensis*). A total of 88 sequences were obtained from the *R. amethystoides* rhizospheric soil, of which eight phylotypes belonged to the genus *Bacillus*, and the others to the genus *Brevibacterium* (Supplementary Figure 1). The number and alpha diversity (Shannon–Wiener and Simpson’s diversity) index of *Bacillus* in rhizospheric soil of *A. adenophora* were significantly higher than those of *R. amethystoides* [numbers: *F*_(1,4)_ = 29.952, *p* = 0.005; Shannon–Wiener index: *F*_(1,4)_ = 28.491, *p* = 0.006; Simpson’s diversity index: *F*_(1,4)_ = 18.424, *p* = 0.013; Table 1]. Among them, seven *Bacillus* phylotypes were found in the rhizospheric soil of both plants; their relative abundance (RA) in the *A. adenophora* rhizospheric soil was as follows: *B. idriensis* 35.25%, *Bacillus toyonensis* 18.03%, *Bacillus thuringiensis* 9.84%, *B. cereus* 7.38%, *Bacillus mycoides* 7.38%, *Bacillus simples* 2.46%, and *B. frigoritolerans* 5.74%, whereas the RA in the *R. amethystoides* rhizospheric soil was as follows: *B. idriensis* 4.55%, *B. toyonensis* 22.73%, *B. thuringiensis* 31.82%, *B. cereus* 4.55%, *B. mycoides* 2.27%, *B. simples* 15.91%, and *B. frigoritolerans* 15.91%. A number of nine *Bacillus* phylotypes were separated only from the rhizospheric soil of *A. adenophora* (*B*. *pumilus* 3.28%, *B*. *licheniformis* 0.82%, *Bacillus tequilensis* 3.28%, *L*. *xylanilyticus* 0.82%, *S*. *aquimarina* 0.82%, *S*. *isronensis* 0.82%, *Bacillus subterraneus* 0.82%, *Bacillus firmus* 2.46%, and *Bacillus anthracis* 0.82%). A number of two *Bacillus* phylotypes were separated only from the rhizospheric soil of *R. amethystoides* (*Bacillus thioparans* 1.14%, *Bacillus wiedmannii* 1.14%) (Figure 1). All of the above strains except *Bacillus anthracis* have been reported to have a strong plant growth-promoting ability.

**TABLE 1:** The number and diversity of *Bacillus* in different treatments.

	AB				RB			
	In	Am	A+R	Rm	In	Am	A+R	Rm
Number of *Bacillus* (CFU/g Soil)	114.333 ± 7.094d**^†^**	64.667 ± 2.517b**^†^**	80.000 ± 4.000c**^†^**	34.000 ± 7.810a**^†^**	86.667 ± 5.132c	38.000 ± 2.000a	47.000 ± 4.359ab	55.333 ± 5.859b
Shannon-Wiener index	2.081 ± 0.101c**^†^**	1.683 ± 0.035ab	1.794 ± 0.087b	1.576 ± 0.031a**^†^**	1.749 ± 0.038c	1.642 ± 0.040b	1.650 ± 0.043bc	1.320 ± 0.031a
Simpson’s diversity index	0.829 ± 0.014b**^†^**	0.731 ± 0.021a**^†^**	0.735 ± 0.023a**^†^**	0.740 ± 0.019a	0.779 ± 0.014b	0.776 ± 0.014b	0.773 ± 0.154b	0.718 ± 0.016Aa

*Shannon–Wiener and Simpson’s diversity index: used to reflect alpha diversity which reflects the species diversity of Bacillus. AB, Bacillus in A. adenophora rhizospheric soil; RB, Bacillus in R. amethystoides rhizospheric soil. In, initial inoculum, Am, A. adenophora monoculture; A+R, A. adenophora and R. amethystoides mixture; Rm, R. amethystodies monoculture. Different lowercase letters indicate significant differences among the four different treatments (three plant growth treatments and initial inoculum) at p < 0.05. ^†^Represent significant differences between the Bacillus of A. adenophora and R. amethystoides with in the same treatment.*

#### *Bacillus* Community Structure in Different Plant Growth Treatments After the Pot Experiment

Compared to the initial inoculum of *A. adenophora* and *R. amethystoides*, the number and Shannon–Wiener index of *Bacillus* in rhizospheric soil of different treatments were significantly decreased after the pot experiment [*A. adenophora*: numbers: *F*_(3,8)_ = 100.179, *p* < 0.001; Shannon–Wiener index: *F*_(3,8)_ = 28.558, *p* < 0.001; *R. amethystoides*: numbers: *F*_(3,8)_ = 64.238, *p* < 0.001; Shannon–Wiener index: *F*_(3,8)_ = 70.436, *p* < 0.001; Table 1]. For the inoculants of *A. adenophora*, Shannon–Wiener index of A+R was significantly higher than that of Rm (*p* < 0.05), whereas there was no significant difference in Simpson’s diversity index among the three treatments (Table 1). A total of three *Bacillus* taxa with high relative abundance were selected as the dominant bacteria. *B*. *idriensis* (41.54%), *B*. *toyonensis* (21.54%), and *B*. *cereus* (12.31%) were dominant in Am; *B*. *idriensis* (40.00%), *B*. *toyonensis* (23.75%), and *B*. *cereus* (10.00%) were dominant in A+R; and *B*. *idriensis* (36.36%), *B*. *thuringiensis* (21.21%), and *B*. *frigoritolerans* (18.18%) were dominant in Rm (Figure 1). For the inoculants of *R. amethystoides*, the alpha diversity index of Rm was lowest compared to Am and A+R (all *p* < 0.01; Table 1). *B*. *toyonensis* (28.95%), *B*. *thuringiensis* (21.05%), and *B*. *simples* (21.05%) were dominant in Am; *B*. *toyonensis* (26.67%), *B*. *thuringiensis* (24.44%), and *B*. *frigoritolerans* (22.22%) were dominant in A+R; and *B*. *thuringiensis* (35.29%), *B*. *simples* (23.53%), and *B*. *frigoritolerans* (23.53%) were dominant in Rm (Figure 1).

### Total Biomass

**FIGURE 2:**
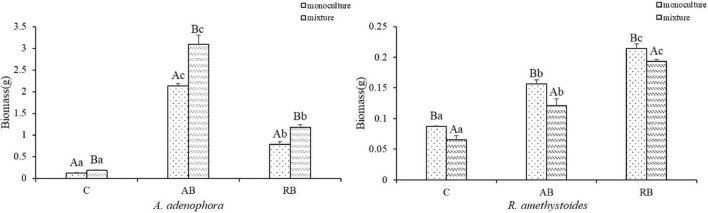
Effect of *Bacillus* on the plant dry biomass of invasive plant *A. adenophora* and native plants *R. amethystoides* when grown in monoculture or in mixture. C, control treatment; AB, *Bacillus* in *A. adenophora* rhizospheric soil; RB, *Bacillus* in *R. amethystoides* rhizospheric soil. Different lowercase letters indicate significant differences among the inoculation treatments at *p* < 0.05. Different capital letters indicate significant differences between the monoculture and mixture at *p* < 0.05. Error bars represent ± 1SE of the mean (*n* = 5).

The effects of inoculation with *Bacillus* from *A. adenophora* (AB) and *R. amethystoides* (RB) were different on the biomasses of *A. adenophora* and *R. amethystoides* (Figure 2). Compared to the uninoculated treatment, the biomasses of *A. adenophora* and *R. amethystoides* were significantly increased by the two kinds of inoculum (*A. adenophora*, monoculture: *F*_(2, 12)_ = 2366.343, *p* < 0.001; mixture: *F*_(2, 12)_ = 666.172, *p* < 0.001; *R. amethystoides*, monoculture: *F*_(2, 12)_ = 565.635, *p* < 0.001; mixture: *F*_(2, 12)_ = 343.127, *p* < 0.001). For *A. adenophora*, the biomass values of inoculated AB treatments in the respective monoculture and mixture were 17.63 and 16.42 times those of the uninoculated treatments, and RB treatments in the respective monoculture and mixture were 6.51 and 6.29 times those of the uninoculated treatments. For *R. amethystoides*, the biomass values of inoculated AB treatments in the respective monoculture and mixture were 1.79 and 1.86 times those of the uninoculated treatments, and RB treatments in the respective monoculture and mixture were 2.46 and 2.97 times of those of the uninoculated treatments. The competition had a favorable effect on the biomass of *A. adenophora* but negatively affected the biomass of *R. amethystoides.* Specifically, the biomass of *A. adenophora* in the mixture was significantly higher than that in the monoculture (monoculture: C:0.121 ± 0.009, AB: 2.133 ± 0.056, RB: 0.788 ± 0.058; mixture: C:0.188 ± 0.006, AB: 3.087 ± 0.213, RB: 1.182 ± 0.057) [C: *F*(_1, 8)_ = 167.773, *p* < 0.001, AB: *F*_(1, 8)_ = 93.638, *p* < 0.001, RB: *F*_(1, 8)_ = 115.269 *p* < 0.001]. In comparison, the biomass of *R. amethystoides* under mixed planting was substantially lower than that in the monoculture (monoculture: C:0.087 ± 0.001, AB: 0.156 ± 0.007, RB: 0.214 ± 0.007; mixture: C:0.065 ± 0.006, AB: 0.121 ± 0.011, RB: 0.193 ± 0.003) [C: *F*_(1, 8)_ = 51.041, *p* < 0.001, AB: *F*_(1, 8)_ = 36.207, *p* < 0.001, RB: *F*_(1, 8)_ = 34.374, *p* < 0.001].

### Corrected Index of Relative Competition Intensity

**FIGURE 3:**
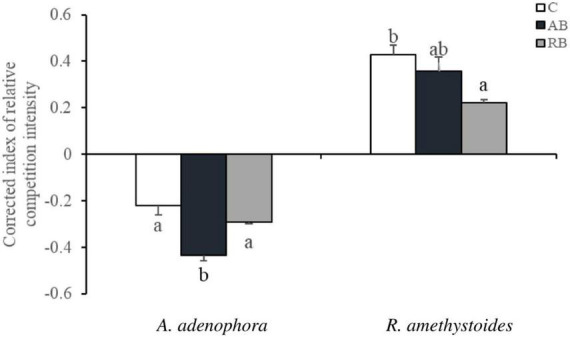
Effect of *Bacillus* on the corrected index of relative competition intensity (CRCI) of invasive plant *A. adenophora* and native plant *R. amethystoides.* Different lowercase letters indicate significant differences among the inoculation treatments at *p* < 0.05. Error bars represent ± 1SE of the mean (*n* = 5).

The CRCI was determined to quantify the effects of *Bacillus* from the two types of rhizospheric soil on the growth of *A. adenophora* and *R. amethystoides*. In all treatments, the competition had a beneficial effect on *A. adenophora* growth and a detrimental effect on *R. amethystoides* growth. Distinct microbial inocula had different effects on the competitive growth of the two plants. The beneficial effect of AB on *A. adenophora* growth resulted in a significant improvement [*F*_(2, 12)_ = 16.096, *p* = 0.001] in which the CRCI of the inoculated AB was two times as high as that of the uninoculated treatment, whereas RB inoculation had no significant effect on the competitive growth. The detrimental impact of RB on *R. amethystoides* growth resulted in a significant reduction [*F*_(2,12)_ = 6.258, *p* = 0.013], whereas AB inoculation did not affect the competitive growth of *R. amethystoides* (Figure 3).

### Total C, N, P, and K Concentrations

**FIGURE 4:**
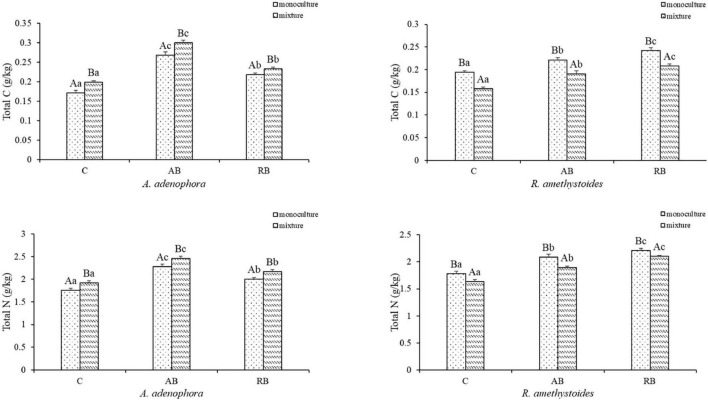
Effect of *Bacillus* on plant total carbon and nitrogen concentrations of invasive plant *A. adenophora* and native plant *R. amethystoides* when grown in monoculture or in mixture. Different lowercase letters indicate significant differences among the inoculation treatments at *p* < 0.05. Different capital letters indicate significant differences between the monoculture and mixture at *p* < 0.05. Error bars represent ± 1SE of the mean (*n* = 5).

**FIGURE 5:**
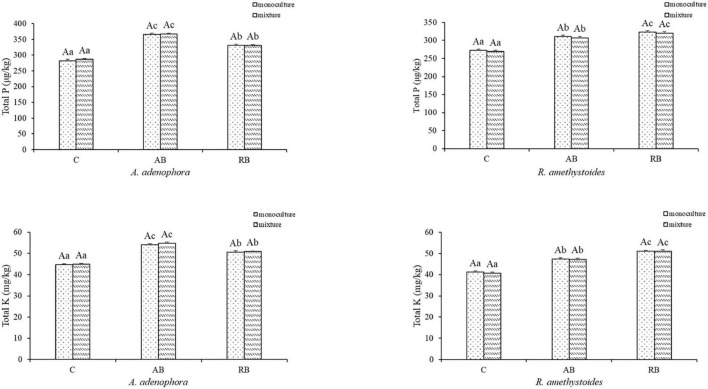
Effect of *Bacillus* on plant total phosphorus and potassium concentrations of invasive plant *A. adenophora* and native plant *R. amethystoides* when grown in monoculture or in mixture. Different lowercase letters indicate significant differences among the inoculation treatments at *p* < 0.05. Different capital letters indicate significant differences between the monoculture and mixture at *p* < 0.05. Error bars represent ± 1SE of the mean (*n* = 5).

Inoculation with *Bacillus* from both rhizospheric soils significantly increased the total C, N, P, and K concentrations of *A. adenophora* and *R. amethystoides* in the monoculture and mixture [*A. adenophora*: total C: monoculture: *F*_(2, 12)_ = 288.193, *p* < 0.001; mixture: *F*_(2, 12)_ = 523.010, *p* < 0.001; total N: monoculture: *F*_(2, 12)_ = 149.670, *p* < 0.001; mixture: *F*_(2, 12)_ = 15.549, *p* < 0.001; total P: monoculture: *F*_(2, 12)_ = 527.826, *p* < 0.001; mixture: *F*_(2, 12)_ = 1758.326, *p* < 0.001; total K: monoculture: *F*_(2, 12)_ = 355.287, *P* < 0.001; mixture: *F*_(2, 12)_ = 560.950, *p* < 0.001; *R. amethystoides*: total C: monoculture: *F*_(2, 12)_ = 108.751, *p* < 0.001; mixture: *F*_(2, 12)_ = 143.196, *p* < 0.001; total N: monoculture: *F*_(2, 12)_ = 120.554, *P* < 0.001; mixture: *F*_(2, 12)_ = 288.873, *p* < 0.001; total P: monoculture: *F*_(2, 12)_ = 305.177, *p* < 0.001; mixture: *F*_(2, 12)_ = 317.306, *p* < 0.001; total K: monoculture: *F*_(2, 12)_ = 507.227, *p* < 0.001; mixture: *F*_(2, 12)_ = 414.302, *p* < 0.001; Figures 4, 5]. The total C, N, P, and K concentrations in *A. adenophora* in the monoculture and mixture were significantly higher in the AB treatment than in the other treatments. Similarly, the total C, N, P, and K concentrations of *R. amethystoides* in the monoculture and mixture with RB treatment were significantly higher than those in the other treatments. Competition had a significant effect on the total C and N concentrations of *A. adenophora* and *R. amethystoides*. However, the total P and K concentrations of the two plants were unaffected. Specifically, when *A. adenophora* was planted in a mixture with *R. amethystoides*, the total C and N concentrations of *A. adenophora* increased compared to monoculture [total C: C: *F*_(1, 8)_ = 73.741, *p* < 0.001, AB: *F*_(1, 8)_ = 44.380, *p* = 0.001, RB: *F*_(1, 8)_ = 31.649, *p* < 0.001; total N: C: *F*_(1, 8)_ = 32.268, *p* < 0.001, AB: *F*_(1, 8)_ = 28.142, *p* = 0.001, RB: *F*_(1, 8)_ = 34.088, *p* < 0.001], but those of *R. amethystoides* decreased [total C: C: *F*_(1, 8)_ = 279.075, *p* < 0.001, AB: *F*_(1, 8)_ = 76.003, *p* = 0.001, RB: *F*_(1, 8)_ = 91.746, *p* < 0.001; total N: C: *F*_(1, 8)_ = 31.588, *p* < 0.001, AB: *F*_(1, 8)_ = 51.322, *p* < 0.001, RB: *F*_(1, 18)_ = 37.579, *p* < 0.001, Figure 4]. However, in the mixed treatment, the total P and K concentrations of *A. adenophora* and *R. amethystoides* were similar to those in the monoculture treatment (Figure 5).

### Soil Characteristics

For the monoculture treatment of *A. adenophora* and *R. amethystoides*, the nutrient contents including NO_3_^–^-N, NH_4_^+^-N, AP and AK in the soil of *A. adenophora* and *R. amethystoides* treated with two kinds of *Bacillus* were considerably higher than those of the control group [*A. adenophora*: NO_3_^–^-N: *F*_(2, 12)_ = 255.633, *p* < 0.001; NH_4_^+^-N: *F*_(2, 12)_ = 62.282, *p* < 0.001; AP: *F*_(2, 12)_ = 65.245, *p* < 0.001; AK: *F*_(2, 12)_ = 405.944, *p* < 0.001; *R. amethystoides*: NO_3_^–^-N: *F*_(2, 12)_ = 317.878, *p* < 0.001; NH_4_^+^-N: *F*_(2, 12)_ = 61.489, *p* < 0.001; AP: *F*_(2, 12)_ = 94.169, *p* < 0.001; AK: *F*_(2, 12)_ = 305.344, *p* < 0.001]. Between the rhizospheric soil *Bacillus* of the *A. adenophora* and *R. amethystoides* treatments, there was no significant difference in the NH_4_^+^-N and AP contents. However, the NO_3_^–^-N content of *A. adenophora* inoculated with AB was significantly higher than that of *A. adenophora* inoculated with RB (*p* < 0.001), whereas the NO_3_^–^-N and AK contents of *R. amethystoides* inoculated with RB were significantly higher than those of *R. amethystoides* inoculated with AB (all *p* < 0.001, Table 2).

**TABLE 2:** Soil characteristics under different treatments.

Treatments		NO_3_^–^-N (μg/g)	NH_4_^+^-N (μg/g)	Available P (μg/g)	Available K (mg/kg)
Initial soil nutrients contents		14.768 ± 0.272	4.076 ± 0.047	4.860 ± 0.046	30.320 ± 0.056
Am	C	13.258 ± 0.174Ba**^†^**	3.584 ± 0.136Aa**^†^**	4.442 ± 0.168Aa**^†^**	28.106 ± 0.363Aa**^†^**
	AB	15.174 ± 0.090Bc**^†^**	4.282 ± 0.085Ab**^†^**	5.281 ± 0.074Ab**^†^**	33.004 ± 0.126Bb**^†^**
	RB	14.230 ± 0.124Ab**^†^**	4.130 ± 0.082Ab	5.166 ± 0.117Ab**^†^**	32.690 ± 0.361Ab**^†^**
A+R	C	14.170 ± 0.116Ca**^†^**	3.534 ± 0.109Aa**^†^**	4.559 ± 0.097Aa**^†^**	29.462 ± 0.252Ba**^†^**
	AB	16.114 ± 0.114Cc**^†^**	4.192 ± 0.116Ab	5.343 ± 0.136Ab**^†^**	33.104 ± 0.379Bb**^†^**
	RB	15.190 ± 0.296Bb**^†^**	4.192 ± 0.059Ab**^†^**	5.289 ± 0.146Ab**^†^**	32.640 ± 0.384Ab**^†^**
Rm	C	12.768 ± 0.167Aa**^†^**	3.582 ± 0.106Aa**^†^**	4.367 ± 0.147Aa**^†^**	28.124 ± 0.269Aa**^†^**
	AB	14.466 ± 0.160Ab	4.260 ± 0.097Ab**^†^**	5.213 ± 0.085Ab**^†^**	31.464 ± 0.290Ab**^†^**
	RB	15.126 ± 0.127Bc**^†^**	4.224 ± 0.122Ab	5.185 ± 0.089Ab**^†^**	32.382 ± 0.300Ac**^†^**

*^†^C, control treatment; AB, Bacillus in A. adenophora rhizospheric soil; RB, Bacillus in R. amethystoides rhizospheric soil; Am, A. adenophora monoculture; A+R, A. adenophora and R. amethystoides mixture; Rm, R. amethystoides monoculture. Different letters in the lower case indicate significant differences between the inoculation treatments at p < 0.05. Different uppercase letters indicate significant differences between the monoculture or mixture at p < 0.05. Daggers represent significant differences between initial soil and after pot experiment soil nutrient contents.*

Inoculation with the two kinds of *Bacillus* significantly increased the contents of available soil nutrients in the mixed treatment of *A. adenophora* and *R. amethystoides* compared to the control treatment [NO_3_^–^-N: *F*_(2, 12)_ = 124.655, *p* < 0.001; NH_4_^+^-N: *F*_(2, 12)_ = 74.726, *p* < 0.001; AP: *F*_(2, 12)_ = 58.227, *p* < 0.001; AK: *F*_(2, 12)_ = 166.378, *p* < 0.001] In the soil of *R. amethystoides*, no significant difference in the NH_4_^+^-N, AP, and AK contents was observed between the two kinds of *Bacillus*. However, the NO_3_^–^-N content of *A. adenophora* inoculated with AB was significantly higher than that of *A. adenophora* inoculated with RB (*p* < 0.001). The content of NO_3_^–^-N in the mixture was higher than that in the monoculture for both plants [*F*_(2, 12)_ = 219.105, *p* < 0.001, Table 2]. This is consistent with the result of the all-strain function experiment. To determine the nitrogen-fixing ability, we performed a functional assay on all isolated strains that were able to survive on Ashby medium. The results showed that the proportion of nitrogen-fixing *Bacillus* in the rhizosphere of *A. adenophora* (the number of nitrogen-fixing strains/total strains, 20.217 ± 1.515%) was significantly higher than that in the rhizosphere of *R. amethystoides* [15.693 ± 1.585%, *F*_(1, 4)_ = 0.004, *p* = 0.023].

## Discussion

The rhizospheric microbial community affects the population establishment of invasive plants in introduced areas (Sun et al., 2021). Our study revealed that the abundance and diversity of *Bacillus* in the rhizospheric soil of *A. adenophora* were more than those of native plants (Table 1 and Figure 1), and the *Bacillus* community in *A. adenophora* rhizospheric soil was beneficial to the competitive growth of *A. adenophora* (Figure 3). Previous study on *A. adenophora* and *Bacillus* has shown that *A. adenophora* could accumulate *B. subtilis* and *B. megaterium* in its invaded areas, whereas the abundance of the two bacteria taxa in the native plant areas is relatively lower (Niu et al., 2007). This study also revealed that the invasion of *A. adenophora* will promote the accumulation of *B. cereus*, which may in turn accelerate the growth of *A. adenophora* (Yang G. Q. et al., 2014; Sun et al., 2021). Here, we used monocultures and mixtures of the invasive plant *A. adenophora* with the native accompanying plant *R. amethystoides*, to demonstrate that inoculum with *Bacillus* could change their competitive growth. We found that the biomass of *A. adenophora* was higher when grown together with *R. amethystoides* in a mixture than grown in a monoculture, and this positive effect was enhanced by inoculating *Bacillus*. After the competitive experiment, the dominant *Bacillus* taxa in the rhizospheric soil of *A. adenophora* were *B. idriensis*, *B. toyonensis*, and *B. cereus* (Figure 1). The results showed that the number and abundance of *Bacillus* in rhizospheric soil of *A. adenophora* were higher than that of native accompanying plants, which promoted the competitive ability and invasion of *A. adenophora*.

The accumulation of beneficial soil bacteria in the rhizosphere of invasive plants increased their nutrient concentration and facilitated their invasion, which in turn provides an indirect advantage for the invasive plants to compete with the native species (Inderjit and van der Putten, 2010; Yu et al., 2021). It has been reported that *Bacillus* inoculated in the rhizospheric soil of plants has a significant growth-promoting function and can promote the absorption of nutrients by plants (Janarthine and Eganathan, 2012; Shameer et al., 2020). For example, inoculation with *B. pumilus* improved the plant N uptake, rhizobacterial population, and further improved plant growth (Marchut-Mikoajczyk et al., 2021). In our study, compared to the *Bacillus* community in rhizospheric soil of *R. amethystoides*, more functional *Bacillus* taxa, such as *B*. *pumilus, B*. *licheniformis, B*. *tequilensis, B*. *firmus, B*. *subterraneus*, and *S*. *aquimarina*, were found in rhizospheric soil of *A. adenophora* (Figure 1). The invasion of *A. adenophora* selected specific microbial taxa in soil, which may have mediated soil nutrient cycling and thus potentially improved plant nutrient acquisition (Xia et al., 2021; Li et al., 2022). Different *Bacillus* taxa vary in their ability to supply plants with nutrients (Pinyapach et al., 2018; Kang et al., 2019). Our results showed that *Bacillus* addition increased the C and N concentrations of *A. adenophora* while grown with *R. amethystoides*, whereas the C and N concentrations of *R. amethystodies* were significantly lower (Figure 4). The leaves of *A. adenophora* have higher CO_2_ fixation capacities, N concentration, and N-use efficiency than those of native accompanying species (Chen et al., 2016). Higher N content of *A. adenophora* improved its competitive ability over the native plant *Lolium perenne* (Zhao et al., 2007). N availability is critical for *A. adenophora* invasion under various soil conditions, and an increasing number of evidence suggests that higher N uptake by invasive plants provides them with a competitive advantage over native species (Jo et al., 2017; Zhang et al., 2020; Chen et al., 2021).

Invasive plants can outbreak in various environments and quickly establish populations as dominant species (Langmaier and Lapin, 2020; Wondafrash et al., 2021). One of the main reasons is that they interact with soil microorganisms to increase the availability of soil resources (Wang et al., 2019). Soil microorganisms have different effects on the nutrient absorption of different plants, thus affecting plant competitiveness (Yang G. W. et al., 2014; Sun et al., 2019). For the *Bacillus* in rhizospheric soil of *A. adenophora*, *B*. *idriensis*, *B*. *toyonensis*, and *B*. *cereus* were dominant in mixture treatment. *B. idriensis* was the most abundant *Bacillus* of *A. adenophora*, which have the potential abilities to promote seedling root growth, desorbing phosphorus and producing indoleacetic acid and ammonia (Afzal et al., 2016; Gholamalizadeh et al., 2017). *B. cereus* enhanced the soil microbial biomass, enzyme activity, N_2_-fixation, and P solubilization (Nayak et al., 2018; Azeem et al., 2021). *B. toyonensis* had direct and indirect plant growth-promoting traits and facilitated plant growth (Rojas-Solis et al., 2020; Zerrouk et al., 2020). The concentration of NO_3_^–^-N in *A. adenophora* soil was higher than that in *R. amethystoides* rhizospheric soil in our study, whether or not *A. adenophora* was grown with native species (Table 2). Among the 20 strains with nitrogen fixation ability, there were seven strains of *B. idriensis*, five strains of *B. toyonensis*, and five strains of *B. cereus*. This reflects the differences in *Bacillus* communities closely linked to soil N transformation (Kuypers et al., 2018). *A. adenophora* may induce higher N-fixation rates by enriching the higher abundance of functional microbes (Zhao et al., 2019; Li et al., 2022). Different nutrient availabilities of rhizospheric soil may lead to asymmetric competition between plant species (Yang G. W. et al., 2014). Therefore, the invasion of *A. adenophora* affected the growth of native plants by increasing or/and shifting its N uptake in the presence of specific *Bacillus* community.

In conclusion, we used the culture-dependent method to detect the positive feedback effect of the rhizospheric *Bacillus* community on *A. adenophora* growth promotion and competition function. Results showed that *A. adenophora* invasion increased the abundance and diversity of *Bacillus* in its rhizospheric soil compared to native accompanying plant rhizospheric soil. *B. idriensis*, *B. toyonensis*, and *B. cereus* might be recruited by *A. adenophora*, and increased the NO3^–^-N content in soil, enhanced C and N concentrations of plant to facilitate the growth and competitiveness of *A. adenophora*. In addition, the culture-dependent method was used in our investigation to detect the phylogenetic structure and diversity of *Bacillus* in the rhizospheric soil of the two plants. Based on our 16S rDNA results, quantity PCR can be performed to more accurately analyze the abundance of *Bacillus* taxa and elucidate the role of *Bacillus* in the invasion process of *A. adenophora* for further studies.

## Data Availability Statement

The original contributions presented in this study are included in the article/Supplementary Material, further inquiries can be directed to the corresponding author/s.

## Author Contributions

FG, ED, and YC designed the research. YL and YC collected the samples. ED performed the experiments. ZS and YL performed the bioinformatic and statistical analyses. ED and YC wrote the first draft. ZS and FG reviewed the manuscript. All authors contributed to the article and approved the submitted version.

## Conflict of Interest

The authors declare that the research was conducted in the absence of any commercial or financial relationships that could be construed as a potential conflict of interest.

## Publisher’s Note

All claims expressed in this article are solely those of the authors and do not necessarily represent those of their affiliated organizations, or those of the publisher, the editors and the reviewers. Any product that may be evaluated in this article, or claim that may be made by its manufacturer, is not guaranteed or endorsed by the publisher.

## Footnotes

1
http://www.ezbiocloud.net
