# Supplementary material for: Rhizospheric Bacillus-Facilitated Effects on the Growth and Competitive Ability of the Invasive Plant Ageratina adenophora
Source: Front Plant Sci. 2022 Jun 14;13:882255. doi: 10.3389/fpls.2022.882255 (PMC9237563; doi:10.3389/fpls.2022.882255)
Supplement: Supplementary file 1 [file Data_Sheet_1.docx]

Supplementary Material

## 1. Supplementary Figures


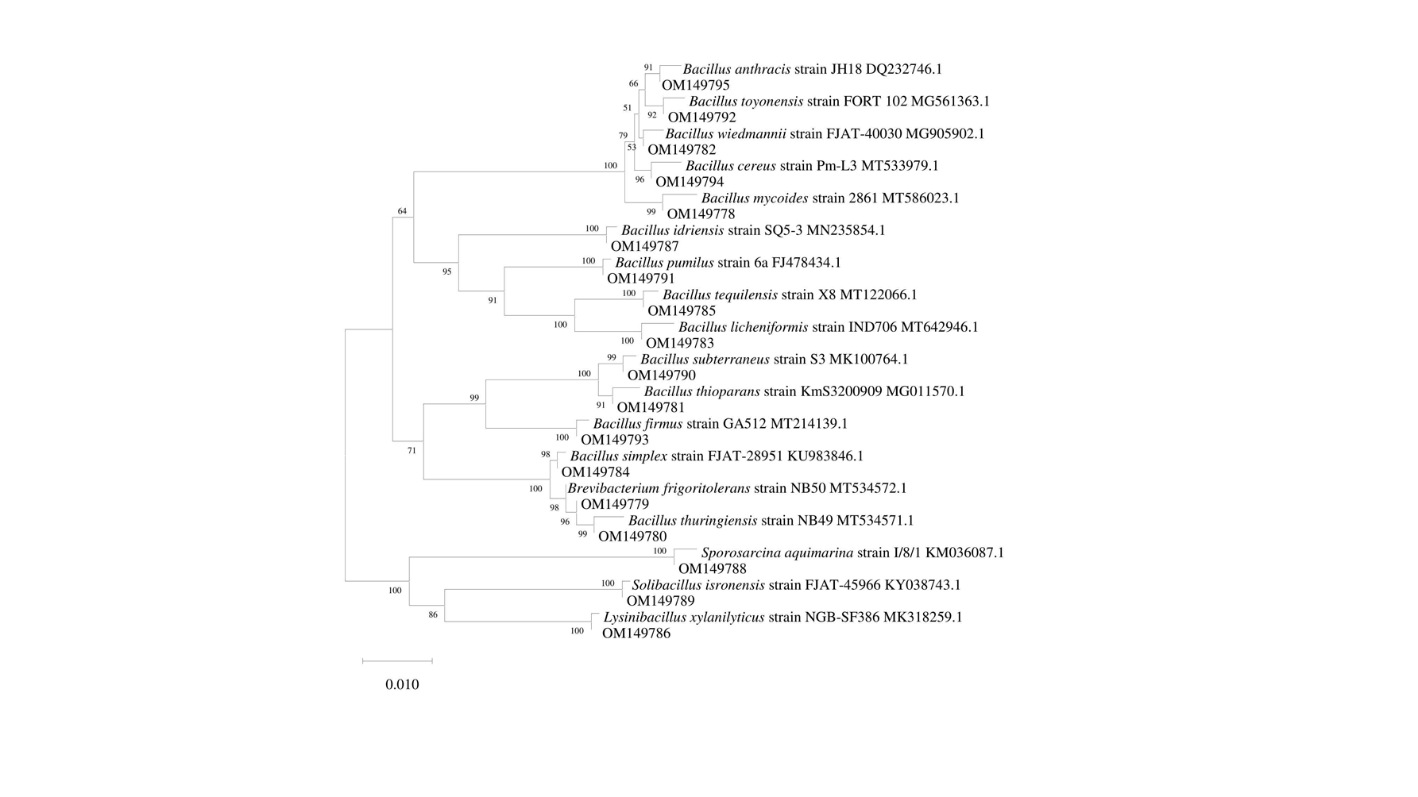


**Supplementary Figure 1.** Neighbor-joining tree showing phylogeny of representative *Bacillus* DNA sequences isolated from rhizosphere of *A. adenophora* and *R. amethystoides* and reference sequences from GenBank. Sequences from this study are identified by their GenBank sequences beginning with OM149. Closely aligned sequences from GenBank are identified by the species name and accession number. The scale bar represents 1% estimated sequence divergence.
